# Differences in Genotype and Antimicrobial Resistance between *Campylobacter* spp. Isolated from Organic and Conventionally Produced Chickens in Sweden

**DOI:** 10.3390/pathogens10121630

**Published:** 2021-12-16

**Authors:** Ingrid Hansson, Patrik Ellström, Oskar Nilsson, Matilda Chaba, Moa Skarin, Lise-Lotte Fernström, Sara Frosth

**Affiliations:** 1 Department of Biomedical Sciences and Veterinary Public Health, Swedish University of Agricultural Sciences, P.O. Box 7036, SE-750 07 Uppsala, Sweden; dodi_95@hotmail.com (M.C.); moa.skarin@slu.se (M.S.); lise-lotte.fernstrom@slu.se (L.-L.F.); sara.frosth@slu.se (S.F.); 2 Department of Medical Sciences, Uppsala University, SE-751 85 Uppsala, Sweden; patrik.ellstrom@medsci.uu.se; 3 Department of Animal Health and Antimicrobial Strategies, National Veterinary Institute (SVA), SE-751 89 Uppsala, Sweden; oskar.nilsson@sva.se

**Keywords:** antimicrobial resistance, broiler, *Campylobacter jejuni*, *Campylobacter coli*, organic, cgMLST, chicken, quinolones, whole-genome sequencing

## Abstract

Antibiotic resistance is a major challenge worldwide and increased resistance to quinolones in *Campylobacter* is being reported. Analysis of antibiotic resistance was performed on 157 *Campylobacter* strains (123 *C. jejuni* and 34 *C. coli*) from conventional and organic chickens produced in Sweden. Susceptibility for tetracycline, ciprofloxacin, erythromycin, nalidixic acid, streptomycin, and gentamycin was determined by microdilution. All 77 isolates from organic chickens were sensitive to all antibiotics, except two *C. jejuni* that were resistant to tetracycline. Of the 80 isolates from conventional chickens, 22.5% of *C. jejuni* and 11.1% of *C. coli* were resistant to quinolones and 5.6% of *C. jejuni* were resistant to tetracycline. Whole-genome sequencing resulted in 50 different sequence types of *C. jejuni* and six of *C. coli.* Nine sequence types were found in both organic and conventional chickens. Two of these (ST-19 and ST-257) included isolates from conventional broilers with different resistance phenotypes to the remaining isolates from conventional and organic broilers. There are management differences between the production systems, such as feed, breed, use of coccidiostats, and access to outdoor area. It is unlikely that quinolone resistance has arisen due to use of antimicrobials, since fluoroquinolones are not permitted in Swedish broiler production.

## 1. Introduction

*Campylobacter* spp. is the most commonly reported bacterial cause of gastrointestinal disease in humans in Europe and many other parts of the world [1,2,3,4,5]. In Sweden, all chicken flocks have been analyzed at slaughter for presence of *Campylobacter* spp. since the Swedish *Campylobacter* program was initiated by the Swedish Poultry Meat Association in 1991. The purpose of the monitoring program is to reduce the number of chickens colonized with *Campylobacter*, starting at farm level. Significantly higher prevalence of *Campylobacter* has been found in samples from chickens in small-scale production systems, such as organic systems, compared with conventionally produced chickens in Sweden [6]. Higher occurrence in organic poultry has also been found in studies in other countries [7,8,9,10]. Possible reasons for the higher occurrence of *Campylobacter* in small-scale production could be less strict hygiene barriers, since such systems often include access to an outdoor arena for the chickens (mandatory in organic production). In addition to outdoor access, organic chickens are a more slow-growing hybrid and have a higher slaughter age (up to 10 weeks), which is another risk factor for *Campylobacter* colonization [11,12,13]. Since production routines differ between conventional and organic systems, it is likely that sources and routes of transmission, and possibly also genotypes and resistance patterns of *Campylobacter*, differ between the production systems. Although many studies of antibiotic resistance in *Campylobacter* isolates from conventional poultry have been performed, there are knowledge gaps regarding antibiotic resistance in *Campylobacter* from organic poultry. No antibiotics are used in organic poultry production, so any differences in antibiotic resistance in *Campylobacter* isolates from conventional and organic poultry operations are of interest. Furthermore, the Swedish Government has set a target for 30% of Swedish agricultural area to be under certified organic production and for 60% of public food consumption to consist of certified organic products by 2030 [14].

Antibiotic resistance has become a major problem worldwide and will be one of the greatest challenges for humans in the future. In 2017, the World Health Organization (WHO) published a global priority list in order to identify, at global level, the most important resistant bacteria for which there is an urgent need for new treatments. Fluoroquinolone-resistant *Campylobacter* was placed in priority group 2, i.e., high priority [15]. An important cause of emergence and spread of resistant bacteria is the use of antibiotics as growth promoters in animals [16,17,18]. Sweden has a low occurrence of antibiotic resistance in an international perspective, most likely due at least partly to the comparatively low (0.3% of flocks in 2020) use of antibiotics in animals and a ban on use of antibiotics as growth promoters since 1986 [19]. Antibiotic resistance in *C. jejuni* from Swedish chickens has varied during the past 20 years and resistance to quinolones, ciprofloxacin, and nalidixic acid has increased to 24% [20]. However, these results are based on analyses of *C. jejuni* isolated from conventional Swedish chickens, while no studies have been published on *Campylobacter* isolates from Swedish organic chickens. A significant increase in resistance to ciprofloxacin and tetracyclines in *C. jejuni* from chickens has been noted in Europe during the past decade. In 2019, extremely high prevalence of resistance to ciprofloxacin was reported in human *Campylobacter* isolates at European Union (EU) level, of 61.5% for *C. jejuni* (*n* = 23,619) and 61.2% for *C. coli* (*n* = 3111). The overall reported resistance to ciprofloxacin among *Campylobacter* isolates from chickens in 2018 was also high or extremely high among the 28 EU member states, 73.5% in *C. jejuni* (*n* = 3519) and 86.7% in *C. coli* (*n* = 339) [21].

The aim of this study was to determine antimicrobial resistance in *Campylobacter* isolated from Swedish chickens and to compare the resistance pattern and sequence types for *Campylobacter jejuni* and *Campylobacter coli* isolated from different production systems (conventional and organic).

## 2. Results

The majority (78%) of 157 randomly selected isolates were identified as *C. jejuni*. A significant difference (*p* = 0.001) was found between species from different production systems, with 89% of the 80 isolates from conventional chickens identified as *C. jejuni* and the remaining (11%) as *C. coli*, while the corresponding proportion of *C. jejuni* and *C. coli* in 77 isolates from organic chickens was 68% and 32%, respectively.

### 2.1. Antibiotic Resistance 

All 52 *C. jejuni* isolates from organic chickens from 15 producers were sensitive to all six antibiotics tested, except two isolates from the same producer that were resistant to tetracycline. Of the 71 *C. jejuni* isolates from conventional chickens, 22.5% (*n* = 16) were resistant to ciprofloxacin and nalidixic acid, and 5.6% (*n* = 4) were resistant to tetracycline. The remaining isolates originating from conventional chickens were sensitive to all six antibiotics tested (Table 1). The difference in resistance among *C. jejuni* isolated from the two production systems was significant regarding ciprofloxacin (*p* = 0.0001) and nalidixic acid (*p* = 0.0001), but not tetracycline (*p* = 1.0).

All 25 *C. coli* isolated from organic chickens were sensitive to all six antibiotics tested, whereas only one (11%) of nine *C. coli* isolated from conventional chickens was resistant to both ciprofloxacin and nalidixic acid, and one isolate was resistant to streptomycin. Otherwise, no other isolate of *C. coli* from conventional chickens showed any resistance to the other antibiotics tested, i.e., erythromycin, gentamycin, and tetracycline (Table 2). No significant difference (*p* = 0.09) in resistance to quinolones was found among *C. coli* from the two production systems.

### 2.2. Campylobacter Genotypes Determined by Whole-Genome Sequencing

**Figure 1:**
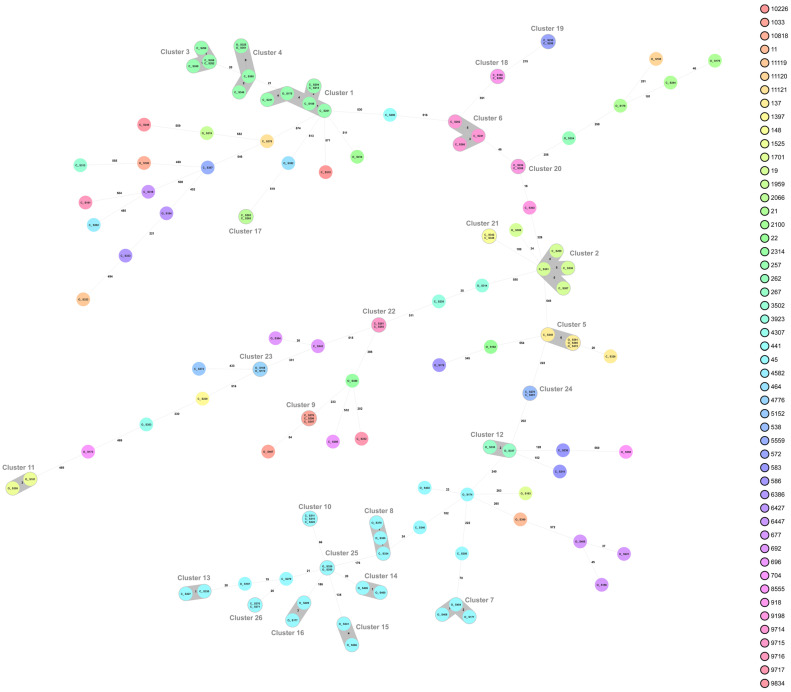
Minimum spanning tree (MST) generated for 121 *Campylobacter jejuni* isolates from organic (O) and conventional (C) chickens in Sweden, based on core genome multi-locus sequence typing (cgMLST) data. MST calculated by pairwise comparison of 637 loci, with missing values ignored. Nodes corresponding to sequenced isolates are colored according to sequence type. Gray background indicates genetically related isolates (maximum difference of 13 cgMLST targets). Values on the lines between nodes represent allelic differences. Line length is not proportional to the numbers.

**Figure 2:**
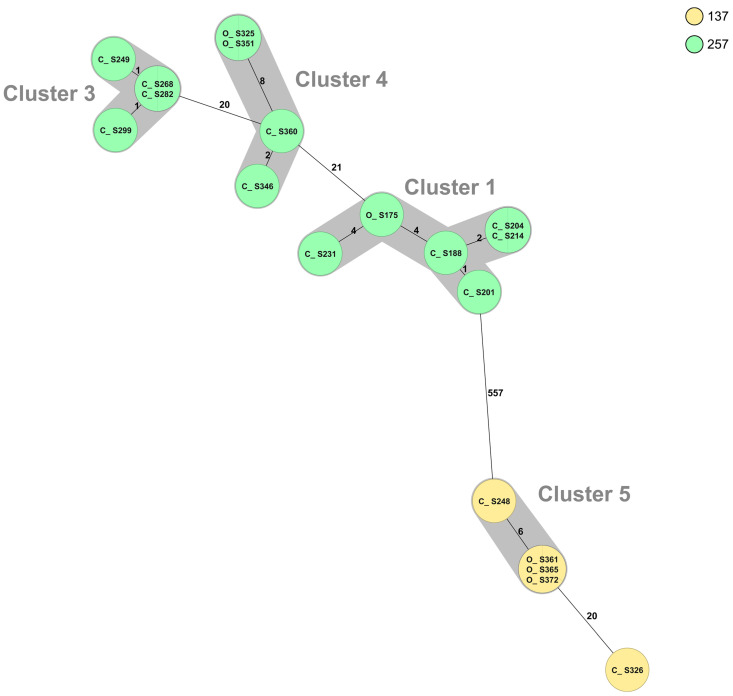
Minimum spanning tree (MST) generated for *Campylobacter jejuni* isolates ST-257 (*n* = 14) and ST-137 (*n* = 5) from organic (O) and conventional (C) chickens in Sweden, based on core genome multi-locus sequence typing (cgMLST) data. MST calculated by pairwise comparison of 637 loci, with missing values ignored. Nodes corresponding to sequenced isolates are colored according to sequence type. Gray background indicates genetically related isolates (maximum difference of 13 cgMLST targets). Values on the lines between nodes represent allelic differences. Line length is not proportional to the numbers.

Whole-genome sequencing (WGS) and multilocus sequence typing (MLST) resulted in 50 sequence types (STs) of *C. jejuni*, with the 52 isolates from organic chickens belonging to 27 different STs and the 69 isolates from conventional chickens to 33 different STs (Table 3). The MLST profile could not be assigned for two *C. jejuni* isolates from conventional chickens. The most common ST of *C. jejuni* was ST-45 (*n* = 27), which were distributed in nine cgMLST clusters and six isolates that did not belong to any cluster (Figure 1). Isolates of ST-257 (*n* = 14) were distributed in three clusters (clusters 1, 3, and 4; Figure 1 and Figure 2). Nine sequence types, (ST-19, ST-21, ST-45, ST-137, ST-257, ST-692, ST-1033, ST-3923, and ST-6427) were isolated from both organic and conventional chickens. The resistance patterns differed between isolates from organic and conventional chickens in two of the sequence types, ST-19 and ST-257. For ST-19, four out of four isolates (cluster 2) from conventional chickens, all originating from different farms, were resistant to quinolones, ciprofloxacin, and nalidixic acid, while the isolate from organic chickens was sensitive to all six antibiotics tested. Two of 11 isolates of ST-257 that originated from two different conventional chicken producers (Figure 2) were resistant to quinolones, ciprofloxacin, and nalidixic acid, while the three isolates of ST-257 from organic chickens were sensitive to all six antimicrobials tested (Table 3). Mutation of T86I in the DNA gyrase (*gyrA*) gene was identified in all 15 (100%) of the quinolone-resistant *C. jejuni* isolates. Of the six isolates with phenotypic resistance to tetracycline, the *tet*(O) gene was identified in five. This gene was also found in three additional isolates that did not show phenotypic resistance.

**Figure 3:**
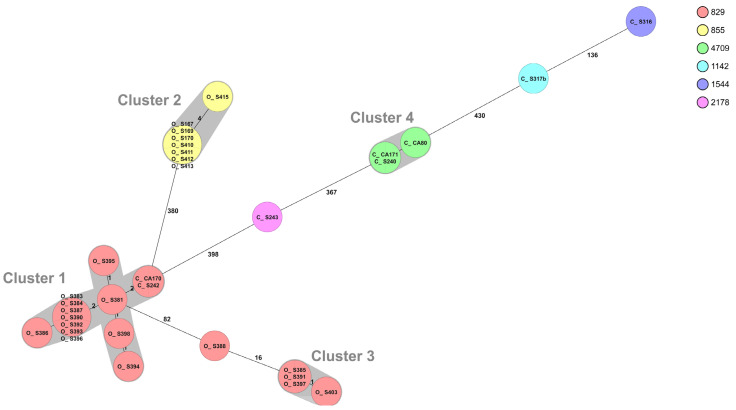
Minimum spanning tree (MST) generated for 33 *Campylobacter coli* isolates from organic (O) and conventional (C) chickens in Sweden, based on core genome multi-locus sequence typing (cgMLST) data. MST calculated by pairwise comparison of 637 loci, with missing values ignored. Nodes corresponding to sequenced isolates are colored according to sequence type. Gray background indicates genetically related isolates (maximum difference of 13 cgMLST targets). Values on the lines between nodes represent allelic differences. Line length is not proportional to the numbers.

The WGS of *C. coli* resulted in six different sequence types, where the 25 isolates from organic chickens belonged to two different STs and the eight isolates from conventional chickens to five different STs (Table 4). The MLST profile could not be assigned for one *C. coli* isolate from conventional chickens. The most common ST among *C. coli* was ST-829 (*n* = 19), and these isolates originated from one conventional broiler producer and two different producers of organic chickens. The two isolates from the conventional producer belonged to cluster 1, together with 12 isolates from one organic producer sampled on different occasions during a six-month period (Figure 3). There are no known connections between the organic and conventional farms, but they are located within about 10 km from each other and there are many deer, wild boar, and birds in the area. All eight strains of ST-855 belonged to the same cluster (cluster 2) (Figure 3) and originated from the same producer of organic chickens. However, they were sampled on eight different sampling occasions during a six-month period.

## 3. Discussion

In this comparison of *Campylobacter* isolates from organic and conventionally raised chickens with respect to species, MLST, and antibiotic resistance profiles, the majority of the 157 isolates selected were identified as *C. jejuni* (78%). This is in line with previous Swedish studies on *Campylobacter* in conventional chickens, reporting 83% and 81% *C. jejuni* [22,23]. *Campylobacter jejuni* isolated from conventionally produced chickens showed a significantly higher occurrence of resistance to the quinolones nalidixic acid and ciprofloxacin compared with *C. jejuni* isolated from organic chickens. This difference between isolates from the different production systems is in agreement with findings in other studies of organic and conventional poultry and poultry meat [24,25,26]. For *C. coli*, the number of isolates was too low to reach statistical power, but the two isolates in which antibiotic resistance was detected were from conventionally produced chickens. Even if the sample sizes appear low, the occurrence of antibiotic resistance among *C. jejuni* isolated from conventional chickens is comparable to that reported in the Swedish monitoring program Svarm [20]. There are no previous reports on antibiotic resistance in *C. coli* isolated from Swedish chickens. In the Svarm report, only *C. coli* from pigs showed documented resistance to quinolones, with 30–40% occurrence during the past 10 years [20]. However, the occurrence of resistance detected in the present study was significantly lower than that reported to the European Food Safety Authority (EFSA) for chicken isolates in other European countries (73.5% for *C. jejuni* and 86.7% for *C. coli*). A high level of resistance has also been observed in isolates from humans in Europe, with average observed resistance to ciprofloxacin in 59.3% of *C. jejuni* isolates and 65.2% of *C. coli* isolates [21]. The practice of antimicrobial usage in poultry production influences the prevalence of antimicrobial-resistant *Campylobacter* organisms in broilers and turkeys, which in turn seems to be reflected in human isolates. The increased resistance to quinolones is considered as a major risk to humans and is observed particularly in countries with frequent treatment of food-producing animals [27,28]. Fluoroquinolone-resistant strains of *C. jejuni* began to be isolated from humans soon after enrofloxacin started to be used in poultry in Europe [29,30]. However, the difference in resistance to quinolones between *Campylobacter* spp. from organic and conventional production systems in Sweden is difficult to explain by selection pressure, as quinolones are not used in any form of poultry production in Sweden. A possible explanation could be the mutable nature of *Campylobacter*. The primary binding sites for quinolones is the gyrase-DNA complex [31] and a point mutation conferring the amino acid substitution T86I in the DNA gyrase (*gyrA*) gene was found in all quinolone-resistant *C. jejuni* in this study. Point mutations in the quinolone resistance-determining region of the *gyrA* gene are known to confer high-level quinolone resistance in *C. jejuni* [32] and most often responsible for resistance to fluoroquinolones [33]. A significant increase in the prevalence of the T86I mutation conferring resistance to fluoroquinolone has been observed in the UK, where the mutation has been found in many different STs [34]. In contrast, a strong association was found between specific genotypes and resistance to antimicrobials in a study on healthy calves in Sweden, with resistance to ciprofloxacin in 66 (46%) of 142 *C. jejuni* isolates [35]. All 20 *C. jejuni* isolates belonging to ST-19 from the calves in that study were resistant to ciprofloxacin and nalidixic acid. In the present study, four out of four *C. jejuni* ST-19 isolates from conventional chickens were resistant to ciprofloxacin and nalidixic acid, while the ST-19 isolate from organic chickens was sensitive to both these antibiotics. Similarly, the *C. jejuni* isolate of ST-441, in this study and that on Swedish calves [35], was resistant to ciprofloxacin, nalidixic acid, and tetracycline, and isolates of ST-21 were sensitive to all antimicrobials tested in both studies. Other studies have also found that resistance to quinolones may be linked to certain sequence types and disseminated among different animal species [36]. However, in the present study two of the 11 ST-257 from conventional chickens and none of the three isolates from organic chickens were resistant to ciprofloxacin and nalidixic acid, whereas in the study on Swedish calves ST-257 isolates were sensitive to all antibiotics tested [35]. It is unclear why *Campylobacter* strains isolated from organic chickens were resistant to ciprofloxacin and nalidixic acid to a significantly lower extent than those from conventionally raised chickens. Organic chickens are kept outdoors during the summer time, which is a season with high levels of *Campylobacter* among most animal species in the surroundings [37,38,39], providing potential exposure to many different strains, including resistant strains. Similar differences in the occurrence of resistance between *C. jejuni* from organic and conventionally produced chickens have been observed in an American study, where 2% of *Campylobacter* strains isolated from organically raised poultry were resistant to fluoroquinolones, while 46% and 67% of *Campylobacter* isolates from conventionally raised broilers and turkeys, respectively, were resistant to fluoroquinolones [26]. However, in another American study no significant differences were observed in the prevalence of antimicrobial resistance between isolates collected from organic and conventional production except for tetracycline, for which isolates from organic production had a higher occurrence (82%) of resistance compared with conventional isolates (65%). However, in the latter study 75% of the isolates were resistant to at least one of the antimicrobials tested [38]. In a Danish study, considerable variation in the occurrence of nalidixic acid resistance in *Campylobacter* (ranging between 0 and 100% of isolates) was found between different farms [39]. That study also found that resistant clones of *Campylobacter* continued to persist in the flocks during several rotations although the corresponding antimicrobial agents were not used [39]. The lack of resistance to erythromycin found in this study is in agreement with previous results for *C. jejuni* in chickens in Sweden and Europe [20,21].

One major difference between organic and conventional chicken production is the use of narasin as a coccidiostat in conventional chickens. Narasin also inhibits the growth of various bacterial species, and it has been shown that bacteria in the normal microbiota of poultry, i.e., enterococci, can develop resistance to narasin [40]. It has also been shown that treatment of chickens with coccidiostats can be associated with increased resistance to other antimicrobials in *Campylobacter* isolates from such flocks [41]. Feed additives such as narasin are not used in organic chickens in Sweden, and instead organic producers try to limit the burden of coccidiosis by moving the chickens to a different area at a specific age. Future studies should explore the potential effects of coccidiostats on antimicrobial resistance in *Campylobacter* spp. and the potential mechanisms involved. There were no significant differences between the number of *C. jejuni* STs found in conventional and organic chickens in this study (33/68 vs. 27/49; *p* = 0.6). However, only nine of the 50 STs were shared between the two production systems. According to Han and colleagues, both diet and type of breed influence the colonization of *C. jejuni* in broiler chickens [42]. However, further studies are needed to determine whether local immunity and the microbiota in the intestines can influence the outcome of colonization by different sequence types of *C. jejuni* in chickens.

## 4. Materials and Methods

### 4.1. Broiler Population

Around 100 million chickens are produced every year in Sweden, of which approximately 99% are produced conventionally by members of the Swedish Poultry Meat Association (SPMA). The SPMA covers the entire procedure of production from hatchery to slaughter, and issues rules and guidelines regarding the production of chickens. Due to the SPMA rules on biosecurity and hygiene barriers, organic chicken producers cannot be members of SPMA. Organic chickens have a longer production period and lower stocking density compared with conventional chickens. Furthermore, the number of chickens produced on each farm is lower than on conventional chicken farms (Table 5). From May to September and with optimal weather conditions, organic chickens must have access to an outdoor area for at least 12 h a day. The outdoor area must be a maximum of 150 m away from the broiler house, safe for the birds, and contain trees and bushes. Between the different flock rotations, the outdoor area must have an empty period [43].

Organic broiler chickens are fed organic feed and at least 50% of the feed must be produced on the farm. The feed may contain up to 5% of conventional protein feed originating from agriculture or up to 10% if the conventional feed is not from agriculture (e.g., fish meal) [43]. Organic chickens are slower-growing breeds (those used in Sweden are Rowan Ranger and Hubbard) [44]. The organic feed must be free from antibiotics, coccidiostats, and genetically modified crops [43]. Instead of using coccidiostats, organic broiler chickens can be vaccinated against some coccidial species at an early age, but such vaccines are not often used in Sweden. Instead, organic farmers change the indoor production area when the chickens are three weeks old. 

### 4.2. Sampling and Bacteriological Analysis

A total of 157 *Campylobacter* isolates collected during 2017, 2018, and 2019 from cecum samples from 80 chicken flocks from 15 conventional producers and 77 chicken flocks from 15 organic producers within the Swedish *Campylobacter* program [45]. The organic producers represented all 15 producers included in the program, whereas the 15 conventional producers were selected because they have often delivered broilers colonized with *Campylobacter* to slaughter. Ten intact ceca from 10 chickens in a slaughter batch were collected after scalding and defeathering, but before washing and cooling of carcasses. The ceca were placed in plastic jars without transport medium and sent by regular mail, at ambient temperature, to the National Veterinary Institute, Uppsala, where they were analyzed as one pooled sample according to ISO10272-1 (2017). In brief, pooled cecum contents were directly cultured on modified charcoal cefoperazone deoxycholate agar (mCCDA) by taking a loopful of sample. The mCCDA plates were incubated at 41.5 ± 0.5 °C for 48 ± 4 h in a microaerobic atmosphere generated by the Anoxomat system (Mart BV, Lichtenvoorde, Netherlands). Suspected Campylobacter colonies were confirmed and identified to species level by matrix-assisted laser desorption/ionization time-of-flight mass spectrometry (MALDI-TOF MS). The isolates were irradiated with laser UV light, which broke the molecules in the bacteria into fragments that were thrown towards a detector. The time it took for the fragments to reach the detector were measured. The molecules gave rise to many fragments and a characteristic mass spectrum, which were compared with stored mass spectra of known bacteria in the database (Bruker Daltonics, Billerica, MA, USA). All *Campylobacter* isolates identified were stored in Brain Heart Infusion (BHI) broth (CM1135; Oxoid, Basingstoke, UK) with 15% glycerol at −70 °C. 

### 4.3. Antibiotic Susceptibility Testing 

Susceptibility to selected antibiotic substances was assessed with Sensititre^TM^ EU Surveillance *Campylobacter* EUCAMP2 Plate (ThermoFisher Scientific; Waltham, MA, USA), determining the antibiotic minimum inhibitory concentration (MIC) by broth microdilution following the standards of Clinical and Laboratory Standards Institute [46]. The EUCAMP2 Plate was chosen as it is designed to meet the criteria of the harmonized monitoring of *Campylobacter* from animals in EU (2013/652/EU). Reference strains of *C. jejuni* (CCUG 33560) were used as controls. Epidemiological cut-off (ECOFF) values for determining susceptibility were obtained from the European Committee on Antimicrobial Susceptibility Testing (EUCAST, https://www.eucast.org/mic_distributions_and_ecoffs, accessed on 17 May 2021). The ECOFF values classify isolates with acquired reduced susceptibility as ‘non-wild type’. In this paper, non-wild type isolates are called ‘resistant’, in agreement with the Swedish Veterinary Antibiotic Resistance Monitoring report [20]. This classification is relevant for monitoring purposes, and they are for example used in the harmonized monitoring of campylobacter from animals in EU (2013/652/EU), but it should be understood that resistance defined in this manner does not always refer to clinical resistance.

### 4.4. Whole-Genome Sequencing

Whole-genome sequencing was successfully performed on 154 *Campylobacter* isolates in total; 121 *C. jejuni* and 33 *C. coli.* All isolates were subcultured twice on horse blood agar plates (SVA B341180; National Veterinary Institute, Uppsala, Sweden) for 48 h at 41.5 °C in a microaerobic atmosphere and from single colonies to reduce the risk of contamination. DNA was prepared by magnetic-particle technology using an EZ1 Advanced XL instrument (Qiagen, Hilden, Germany) and the EZ1 DNA Tissue Kit (Qiagen, Hilden, Germany) according to the manufacturer’s instructions for Gram-negative bacteria and the bacterial protocol. The selected elution volume was 100 µL and the Qubit ds DNA High Sensitivity Assay Kit (Invitrogen, Carlsbad, CA, USA) was used on a Qubit^®^ 2.0 Fluorometer (Invitrogen, Carlsbad, CA, USA) to measure the DNA concentration. Sample libraries for sequencing were prepared using the Nextera XT DNA Library Preparation Kit (Illumina, San Diego, CA, USA) according to the manufacturer’s instructions. Prepared libraries were quantified using the Qubit ds DNA High Sensitivity Assay Kit (Invitrogen, Carlsbad, CA, USA) on a Qubit^®^ 2.0 Fluorometer (Invitrogen, Carlsbad, CA, USA) and the quality was checked using the High Sensitivity DNA ScreenTape Analysis D1000 (Agilent Technologies, Inc., Santa Clara, CA, USA) on the 4150 TapeStation System (Agilent Technologies, Inc., Santa Clara, CA, USA). Sequencing of the libraries was performed using the NextSeq 500/550 Mid Output kit V2.5 with 2 × 150-bp paired-end reads (Illumina Inc., San Diego, CA, USA) on an Illumina NextSeq 500 system (Illumina Inc., San Diego, CA, USA).

The resulting sequences were analyzed using the Ridom SeqSphere + v7.0.5 software (Ridom GmbH, Münster, Germany). Genomes were de novo assembled using SKESA [47], through a pipeline script in Ridom SeqSphere+ (Ridom GmbH, Münster, Germany), and MLST profiles were assigned using the scheme at https://pubmlst.org/campylobacter/ accessed on 18 August 2021 [48], also through the Ridom SeqSphere+ (Ridom GmbH, Münster, Germany) software. The *C. jejuni/coli* cgMLST task template v1.3 in Ridom SeqSphere+ (Ridom GmbH, Münster, Germany), which contains 637 loci, was used for core genome MLST (cgMLST) analysis, and to generate a minimum spanning tree (MST). The MST was used to examine the relationship between the isolates. Default parameter settings were used when the MST was constructed and the default value of maximum difference of 13 cgMLST targets was used to designate a relationship. A high agreement between the Ridom SeqSphere+ cgMLST schema with 637 loci and the Oxford cgMLST schema with 1343 loci has recently been shown [49]. New MLST profiles have been deposited in the pubMLST database (https://pubmlst.org/ accessed on 16 August 2021) with isolate identities: 110222–110224. All assembled genomes were analyzed for acquired antimicrobial resistance and chromosomal point mutations specific for *Campylobacter jejuni* and *Campylobacter coli*, respectively, by ResFinder 4.1 [50,51].

### 4.5. Statistical Analysis

The results were analyzed by Fisher’s Exact test, performed using a statistical program on the Internet website “Social Science Statistics” (https://www.socscistatistics.com accessed on 17th May 2021). The tests verified the difference in *Campylobacter* isolated from conventional and organic produced chickens. A probability level of *p* < 0.05 was considered statistically significant.

## 5. Conclusions

*Campylobacter jejuni* isolated from conventionally produced chickens showed significantly higher occurrence of resistance to quinolones than *C. jejuni* isolated from organic chickens. The reasons for this quinolone resistance are not known, but selection through use of antibiotics is unlikely since fluoroquinolones are not used in Swedish broiler production. The differences between the production systems might instead be due to differences in feed, breed, access to outdoor area, and use of coccidiostats. Antimicrobial resistance was detected in 22.5% and 11.1% of *C. jejuni* and *C. coli* isolates, respectively, from conventional Swedish chickens. These levels were significantly lower than in chicken isolates from other European countries, which show resistance rates of 73.5% (*C. jejuni*) and 86.7% (*C. coli*). Sequence typing of the 154 *Campylobacter* strains detected resulted in 56 different STs, of which nine were found in both organic and conventional chickens, suggesting both similarities and differences in colonization ability and/or transmission routes, depending on *Campylobacter* genotype, between the different production systems.

## Figures and Tables

**Table 1:** Distribution of minimum inhibitory concentrations (MICs, mg/L) and antibiotic resistance (Res, %) in *Campylobacter jejuni* isolated from 52 organic and 71 conventional Swedish chicken flocks slaughtered in 2017–2019. The results are shown as percentage of isolates at different MIC values. White fields denote range of dilutions tested for each antibiotic and vertical bold lines indicate cut-off values used to define resistance. MIC values equal to or lower than the lowest concentration tested are given as the lowest concentration tested.

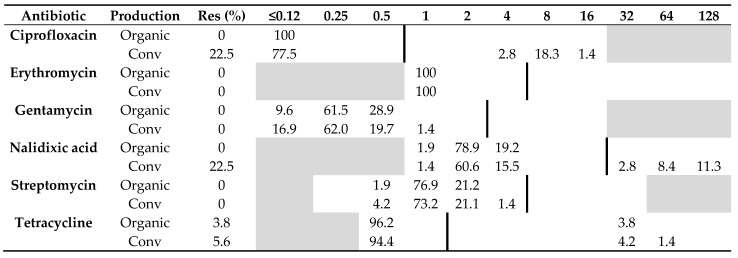

**Table 2:** Distribution of minimum inhibitory concentrations (MICs, mg/L) and resistance (Res, %) in *Campylobacter coli* isolated from organic (*n* = 25) and conventional (*n* = 9) Swedish chickens slaughtered in 2017–2019. The results are shown as percentage of isolates at different MIC values. White fields denote range of dilutions tested for each antibiotic and vertical bold lines indicate cut-off values used to define resistance. MIC values equal to or lower than the lowest concentration tested are given as the lowest concentration tested.

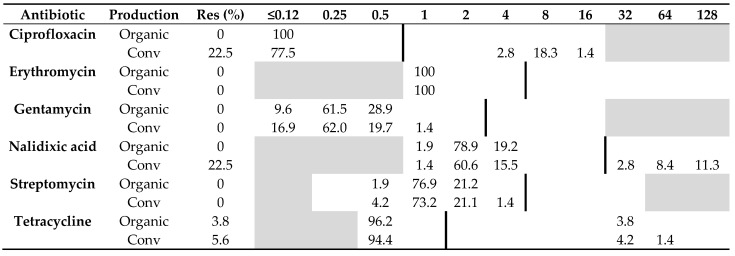

**Table 3:** Distribution of clonal complex (CC) and multi-locus sequence types (MLST), number of resistant isolates, and resistance profile of *Campylobacter jejuni* originating from 52 organic and 69 conventional chicken flocks in Sweden.

		Total	Organic		Conventional	
CC	MLST	No. of Isolates	No. of Isolates	Resistance	No. of Isolates	Resistance ^1^
21	19	5	1	-	4	4 (cip + nal)
21	21	3	2	-	1	-
21	148	2	-	-	2	2 (cip + nal)
21	262	1	1	-	-	-
21	7419	3	-	-	3	3 (cip + nal + tet)
21	9198	3	-	-	3	-
21	11120	1	1	-	-	-
22	22	1	1	-	-	-
45	11	1	1	-	-	-
45	45	27	16	-	11	-
45	137	5	3	-	2	-
45	538	3	-	-	3	-
45	583	2	-	-	2	-
45	1701	1	1	-	-	-
48	918	2	-	-	2	-
52	2066	2	-	-	2	-
52	2100	1	1	1 (tet)	-	-
206	572	2	-	-	2	2 (cip + nal)
257	257	14	3	-	11	2 (cip + nal)
283	267	2	2	-	-	-
464	464	1	1	1 (tet)	-	-
677	677	3	3	-	-	-
692	692	2	1	-	1	-
692	4776	2	2	-	-	-
702	5152	1	-	-	1	-
952	4582	1	-	-	1	-
952	6447	1	-	-	1	-
952	9716	1	-	-	1	-
1034	1033	4	1	-	3	-
1034	1034	1	-	-	1	1 (cip + nal)
1034	2314	1	1	-	-	-
1034	9715	2	-	-	2	-
1332	696	1	-	-	1	-
NA	441	1	-	-	1	1 (cip + nal + tet)
NA	586	1	1	-	-	-
NA	704	1	1	-	-	-
NA	1397	1	-	-	1	-
NA	1525	2	2	-	-	-
NA	1959	1	1	-	-	-
NA	3502	1	-	-	1	-
NA	3923	2	1	-	1	-
NA	4307	1	1	-	-	-
NA	5559	1	-	-	1	-
NA	6386	1	-	-	1	-
NA	6427	1	1	-	-	-
NA	8555	1	1	-	-	-
NA	9834	1	-	--	1	-
NA	10226	1	-	-	1	-
NA	10818	1	1	-	-	-
NA	11119	1	1	-	-	-
NA	11121	1	-	-	1	-

NA = not assigned, ^1^ cip = ciprofloxacin, nal = nalidixic acid, tet = tetracycline.

**Table 4:** Distribution of clonal complex (CC) and multi-locus sequence type (MLST) profiles and antimicrobial resistance of 33 *Campylobacter coli* isolates from organic (*n* = 25) and conventional (*n* = 8) chicken flocks in Sweden.

		Total	Organic		Conventional	
CC	MLST	No. of Isolates	No. of Isolates	Resistant	No. of Isolates	Resistant
82	829	19	17	-	2	-
82	855	8	8	-	-	-
82	1142	1	-	-	1	-
82	1544	1	-	-	1	1 (strept)
82	2178	1	-	-	1	1 (cip + nal)
82	4709	3	-	-	3	-

**Table 5:** Differences in production systems for conventional and organic chickens in Sweden.

	Conventional	Organic
Chicken producers within the Swedish Campylobacter program	110	15
Chickens produced (2017/2018/2019)	100/99/103 million	855,000/660,000/720,000
Chickens in one compartment	Up to 60,000	Up to 4800
Maximum stocking density (kg/m^2^)	36 kg	20 kg
Maximum stocking number (chickens/m^2^)	25	10
Age of slaughter	28–35 days *	60–70 days
Outdoor access	Not at all	May to Sep, >4 m^2^/chicken
Breed	Ross and Cobb	Hubbard, Rowan Ranger
Coccidiostats	Narasin until 3 d before slaughter	Not at all
*Campylobacter* status (2017/2018/2019)	11%/9%/5%	40%/38%/57%

* Except one abattoir slaughtering around 2% of conventional flocks, which has a slaughter age of 50–55 days.

## Data Availability

New MLST profiles have been deposited in the pubMLST database (https://pubmlst.org/ accessed on 17 May 2021) with isolate identities: 110222–110224.
